# Creating Affording Situations: Coaching through Animate Objects

**DOI:** 10.3390/s17102308

**Published:** 2017-10-11

**Authors:** Chris Baber, Ahmad Khattab, Martin Russell, Joachim Hermsdörfer, Alan Wing

**Affiliations:** 1 School of Engineering, University of Birmingham, Birmingham B15 2TT, UK; in06khattab@gmail.com (A.K.); m.j.russell@bham.ac.uk (M.R.); 2 Department of Sport and Health Sciences, Technische Universität Munchen, 80992 Munchen, Germany; joachim.hermsdoerfer@tum.de; 3 School of Psychology, University of Birmingham, Birmingham B15 2TT, UK; a.m.wing@bham.ac.uk

**Keywords:** tangible user interface, affordance, multimodal cueing, animate objects, activities of daily living

## Abstract

We explore the ways in which animate objects can be used to cue actions as part of coaching in Activities of Daily Living (ADL). In this case, changing the appearance or behavior of a physical object is intended to cue actions which are appropriate for a given context. The context is defined by the intention of the users, the state of the objects and the tasks for which these objects can be used. We present initial design prototypes and simple user trials which explore the impact of different cues on activity. It is shown that raising the handle of a jug, for example, not only cues the act of picking up the jug but also encourages use of the hand adjacent to the handle; that combinations of lights (on the objects) and auditory cues influence activity through reducing uncertainty; and that cueing can challenge pre-learned action sequences. We interpret these results in terms of the idea that the animate objects can be used to create affording situations, and discuss implications of this work to support relearning of ADL following brain damage or injury, such as might arise following a stroke.

## 1. Introduction

In this paper we consider ‘coaching’ in terms of encouraging people to act. Broadly, coaching involves a set of processes which are aimed at helping an individual (or group of individuals) improve, develop or learn skills. Often coaching will involve a dialogue between the individual and their coach. Replacing a (human) coach with a digital counterpart, therefore, raises some interesting questions concerning the ways in which to determine the improvement, development or learning by the individual, and the ways in which ‘dialogue’ could occur. Ensuring that the coaching is tailored to the abilities of the individual is essential for digital coaching [1]. From this, a basic specification for a digital coach would include the ability to recognize the actions performed by the individual, to evaluate these actions (against some quality criterion), and to provide advice and guidance that could lead to improvement (or alteration) in the performance of the actions (Table 1).

For example, assume that you will make a hot drink by boiling a kettle and then pouring boiling water into a mug into which you have already added coffee granules. This can be decomposed into a sequence of steps; each step has a set of successive steps which are more likely to lead to the goal. When a person appears confused, e.g., when they fail to act, or when they make an error, i.e., when they perform an action which is not one of the recommended ones, then they might require a prompt. The challenge is to prompt actions in a sequence in order to either correct the sequence, or prevent further errors, and in order not to distract or frustrate the person.

**Figure 1:**
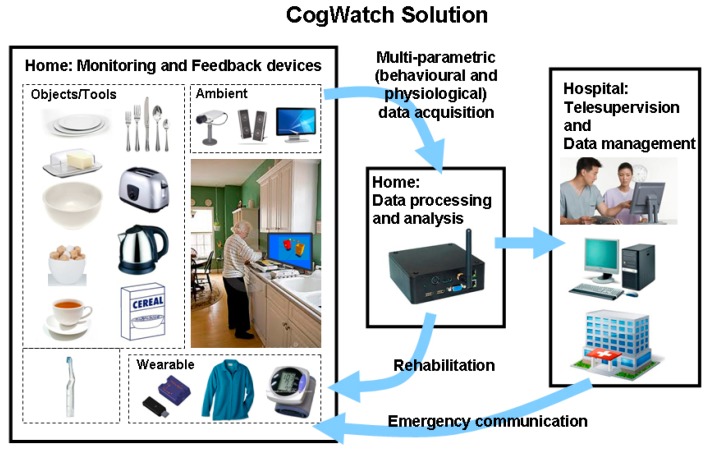
Schematic of the *CogWatch* concept.

Within the EU-funded project *CogWatch* (http://www.cogwatch.eu/, Figure 1) we developed technology that supported Activities of Daily Living (ADL) through recognition of activity and cueing to reduce errors [2,3].

**Figure 2:**
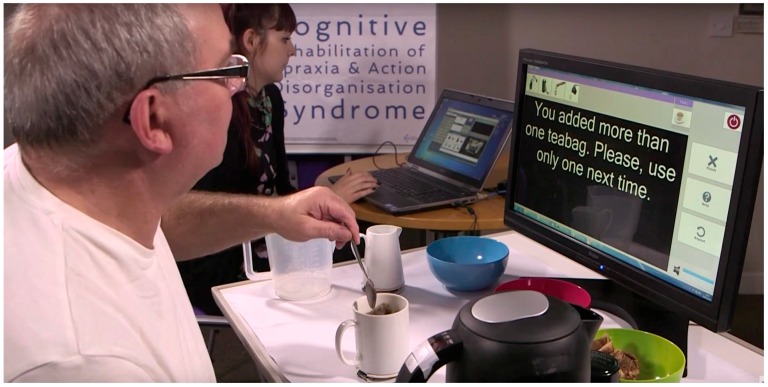
Interacting with the *CogWatch* system.

Figure 2 shows the *CogWatch* system being used. When a person performs a sequence of actions, such as making a cup of tea, each action they perform is recognized and compared with a set of plausible actions. If an action is not part of this plausible set it could be defined as an error, e.g., because an action is repeated or because it was not appropriate at that point in the sequence. If this occurs then the user receives a prompt on the visual display.

Sensors (accelerometers and force sensitive resistors, FSRs) on the objects detect the actions that a person makes with them. Data from the sensors, together with hand tracking using Microsoft Kinect, are used to create Hidden Markov Models for activity recognition [4]. In order to determine when to provide a cue to the user, the activity recognition output is compared with the prediction of the actions which would be appropriate for a goal. The prediction is based on Partially-Observable Markov Decision Process (POMDP) models of task sequence [5,6].

In trials conducted with stroke patients, as part of the *CogWatch* project, it was found that, without support, patients struggle to complete ADL, such as tea-making. Under such conditions, most of the patients tested failing to complete the task successfully. Even when patients were able to consult printed instructions on the step-by-step sequence of actions, they still failed to complete the tasks. We believe that this shows that printed support is ineffective with this particular task and population. Some stroke victims have concomitant cognitive problems in addition to difficulties in executing, and some of these relate to language ability. While efforts were made to select patients with similar impairments, some participants in these trials could have found the printed instructions challenging.

In contrast, almost all of the trials with *CogWatch* support resulted in patients successfully completing the tea-making tasks [2]. However, even in these *CogWatch* trials, patients tended to make errors (which they were able to correct) and took significant time to complete the activity. While the *CogWatch* project demonstrated that patients were able to respond effectively to the cues presented to them, the system relied on the use of a visual display to provide these cues as shown in Figure 2. While Figure 2 shows text instructions (which could create similar problems to those noted for the printed instructions) we also showed guidance using video, and this still led to problems. Another explanation of the time and errors in these trials is that patients might have found it difficult to divide their attention between the physical actions involved in performing the tasks using the objects, and the more abstract task of reading instructions and relating these to their actions. Consequently, in this paper, we explore whether the cues could be provided by the objects themselves.

### Designing Intelligent Objects

Objects can be designed to provide visual, tactile or auditory cues to the user [7]. This develops prior work on Tangible User Interfaces (TUIs) or Ambient Displays [8] which involve the development of ‘smart objects’ [9]. A smart object typically has awareness (defined as the ability to sense where it is, how it is being used etc.), representation (defined as the ability to make sense of its awareness), and interaction (defined as the ability to respond to the user or other objects). In *CogWatch*, as discussed previously, awareness was achieved through the integration of sensors on objects and representation was through the developed on HMM and POMDP. In order to support interaction, we extend the design of these objects to present information to users.

**Figure 3:**
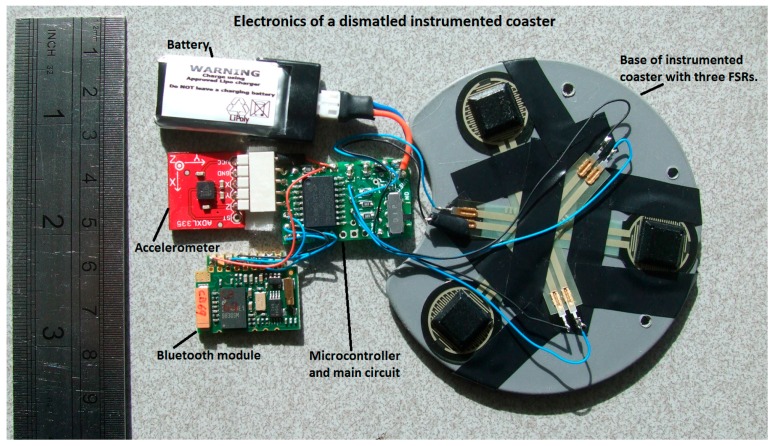
*CogWatch* coaster.

The development of TUIs over the past two decades has been dependent on the availability of miniature sensors and processors. Much of this work has focused on the development of objects as input devices or objects as forms of ambient display. Contemporary work, particularly at MIT [10], has been exploring ways in which objects can be physically transformed. In terms of the healthcare domain, an ambient display has been developed to alert teenagers with Attention Deficit/Hyperactivity Disorder (ADHD) to support everyday activity planning [11]. Similarly, ambient displays can be used to provide reminders to patients concerning the time to take medication [12,13], and a Rehabilitation Internet of Things (RioT) [14] uses wearable sensors to advise on the physical activities of individuals wearing these devices. For *CogWatch*, objects used in ADL were kept as normal as possible in appearance and function, to avoid causing further confusion to the patients. This meant that the sensors had to be small and discrete. For several of the objects used in the archetypical ADL of making a cup of tea, we developed an instrumented coaster. This design allowed us to package the sensors and circuitry (Figure 3). The resulting device can be fitted to the underside of the object, where it has very little visual impact and does not obstruct the use of the object. This is inspired by the well-known MediaCup concept [15].

**Figure 4:**
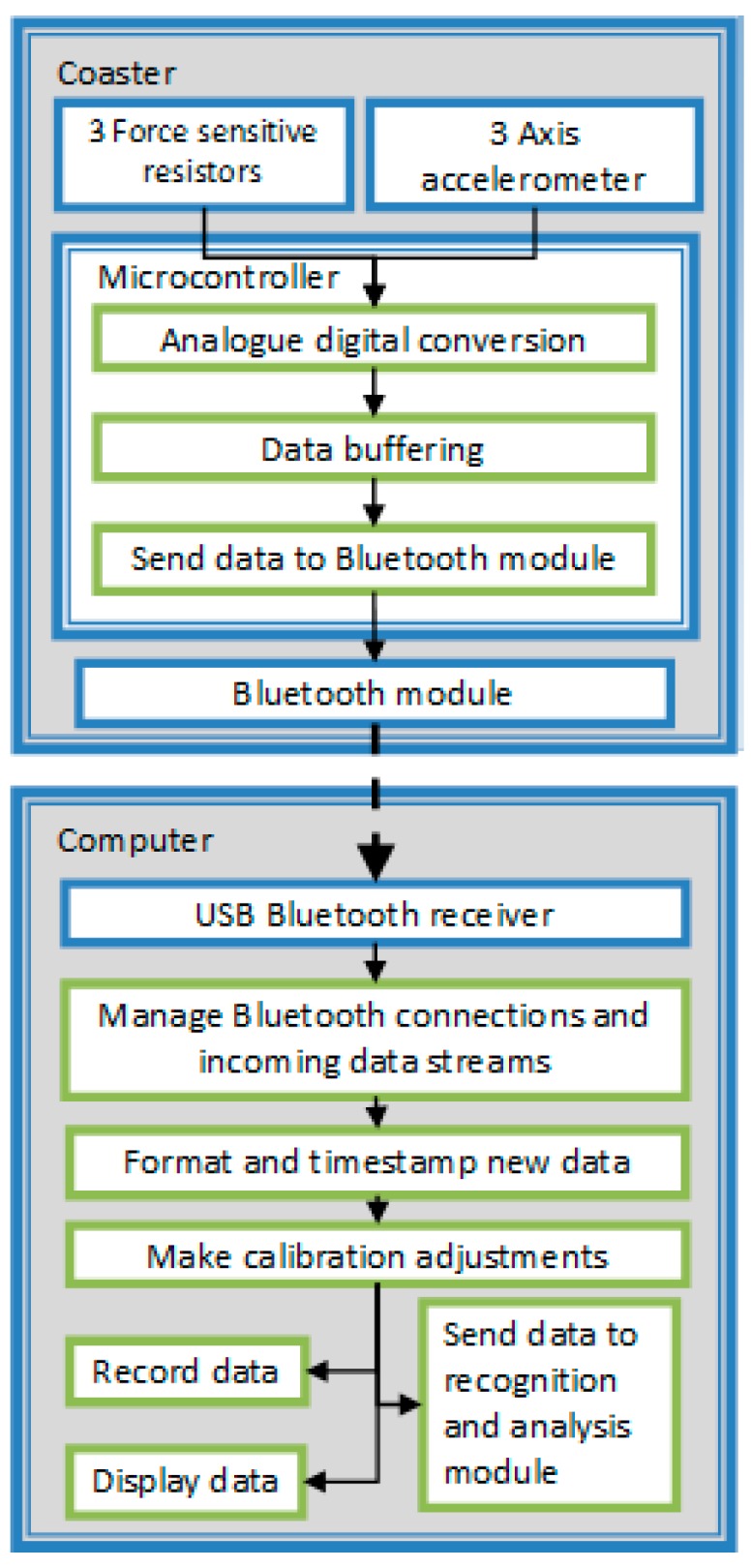
Coaster system design.

The coaster is fitted with Force Sensitive Resistors (FSRs) which are used to not only determine when the object is on the table or lifted, but can also be used to estimate how much liquid is being poured into a container. In addition to FSRs, triaxial accelerometers are used to record movement. The sensors are controlled by a Microchip dsPIC30F3012 microcontroller, which has an integrated 12 bit analogue digital converter (ADC). The microcontroller is programmed to digitize, compress and prepare the sensor data and manage the transmission of the data via Bluetooth. The data are buffered on the microcontroller so that they can be re-transmitted (avoiding data loss) if the wireless connection is interrupted for a short period. An ARF7044 Bluetooth module is used to transmit the sensor data to a host computer via a Bluetooth wireless connection (Figure 4).

As Poupyrev et al. [16] note, there is a tendency for TUIs to respond to users primarily through visual or auditory displays and there has been less work on displays which can change their physical appearance. The development of small, easy-to-use actuators makes it possible for shape-changing objects to be created.

**Figure 5:**
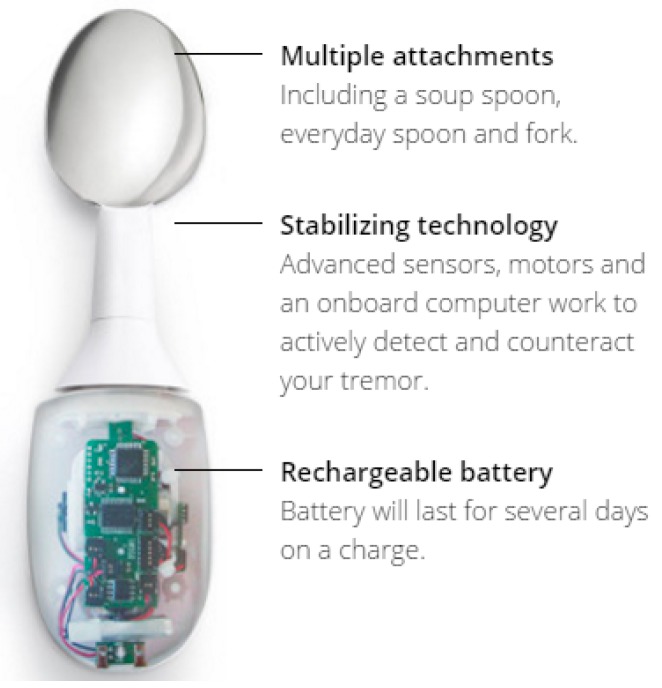
Stabilizing Spoon (https://www.liftware.com/).

In addition to cueing when to perform an action, it is possible to influence the ongoing performance of an action in order to correct or compensate the manner in which the action is performed. For example, Figure 5 shows a commercial product which is designed to compensate for tremors, such as might arise from Parkinson’s disease.

Having the object change its physical behavior, e.g., through vibration, could cue the user to which object to pick up (by making the object wobble on the table) as well as compensating for the movements performed by the user. For example, we experimented, with an arrow on the lid of a jug which would point to the direction in which the person should move the jug (the arrow was connected to a servomotor driven by a magnetometer which responded to magnets placed on the table or in other objects). In order to indicate the state of an object, one can use visual cues to show its temperature or whether it is turned on or off, or open or closed. For example, one could use a Light-Emitting Diode (LED) in the handle of a drawer to alert a person to this drawer [17]. The light could indicate that this particular drawer contains the saucepan that the person needs for making a sauce.

**Figure 6:**
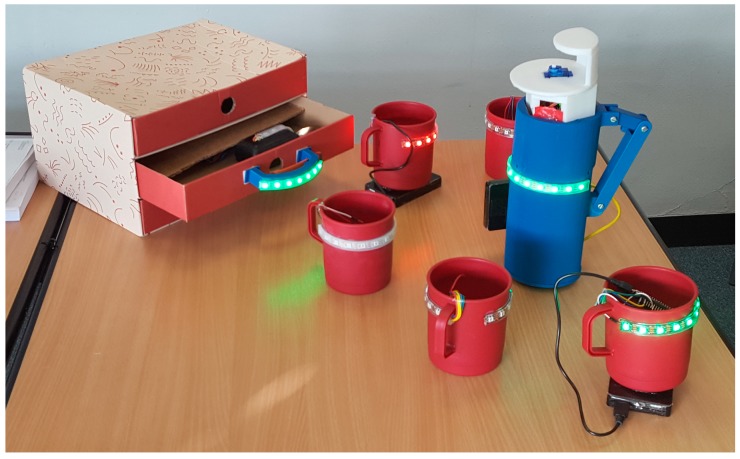
A collection of animate objects.

Figure 6 shows a set of animate objects used in our initial experiments. The jug has an accelerometer and an LED strip. A Force Sensitive Resistor (FSR), on the base of the jug, is used to detect when the jug has been picked up. A tilt sensor, triggered at an angle of 30°, is used to determine when it is poured, and an MP3 player (SOMO II) and speaker are used to play sounds from an SD card. Finally, we reused the laser position assembly from a DVD drive as the mechanism for raising and lowering of the handle of the jug. The spoon has an accelerometer, vibration motor, data logger and an LED strip. The cups have LED strips. All objects connect to the Wi-Fi network (and for these trials, were controlled via an app running on an Android phone).

Note that, in Figure 6, some of the objects have green lights and one mug has red lights. We recognize that green and red may be problematic for color-blind people and this could be reconfigured in later designs. However, in our initial trials we do not tell participants what the lights mean but rather wait to see what interpretation the participants provide. The intention is that the behavior of the objects can both attract the attention of the user and also cues which action to perform. For example, in one trial we place the jug in front of the participant and raise the handle. If the participant does not respond to the handle rising, then the LED strip on the jug turns green. After this, an auditory cue (of the sound of pouring water) is played to draw their attention to the jug and prompt a lift and pour action, and if this fails to elicit a response a verbal prompt (with a voice recording of the phrase ‘pick me up’) is played.

For communications, Wi-Fi was chosen due to its scalability, lack of infrastructure and the ability to control the experiment from a mobile phone. This also allows devices to communicate with each other and change state based on another device’s sensor. All the objects are fitted with an Arduino with built in Wi-Fi (Adafruit Feather M0 Wi-Fi). This Arduino has built in battery management and an SD card shield for data logging. The Wi-Fi server was programmed using Blynk.

**Figure 7:**
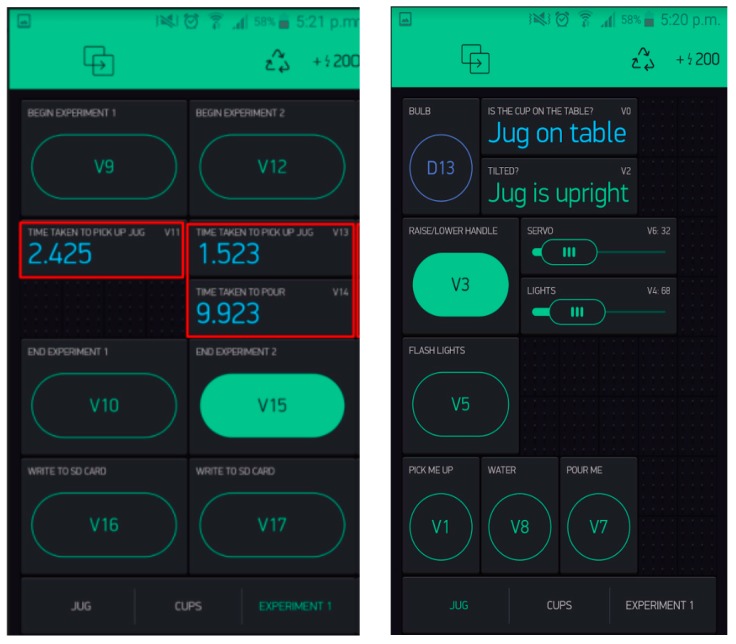
Screenshots of app showing timings for experiments 1 and 2.

Figure 7 shows a user interface, running on an Android mobile telephone that was used to control the objects and record data from the trials. The server’s IP address must remain constant in order to ensure all devices connect to it, but the server must be able to connect to other networks (e.g., the university). This requires a DHCP reservation to be made. The server allows multiple phones to connect so multiple phones can control the experiment, but also provides some security in the connections. A local router could be implemented on a Raspberry Pi Zero which can host both the server and the local network. This raspberry pi is small enough to fit inside the jug which will result in no extra equipment needed other than the jug, spoon, cups. The network enables expansion to other objects.

## 2. Evaluation and Initial User Trials

In this section, we report three small-scale user trials in which participants were asked to perform simple tasks using the animate objects. The aim of this was to explore the ways in which the behavior of the objects could be used to cue actions, and how easily people could interpret and respond to these cues. In these user trials, all participants are neurotypical and were recruited from students in the Engineering School. Thus, we are not considering the question of how patients might react to the objects in these studies.

### 2.1. Participants and Trial Design

All participants gave their informed consent for inclusion before they participated in the study. The study was conducted in accordance with the Declaration of Helsinki, and the protocol was approved by the School of Engineering, University of Birmingham Ethics process. 23 participants (mean age: 25; 19 male, six female). Participants had no prior experience of the equipment or experimental tasks, and received minimal prior instruction as the aim was to see how they would respond on their first encounter with the objects. They were told that the aim of the trials was to allow them to interact with ‘animate objects’ and that they were to perform an action that they believed would be appropriate on an object as soon as they were confident in the opportunity to act. All participants performed three trials, with each trial involving 6–8 repetitions. In total, this produced 650 records, and 7½ hours of video.

The timings, for the first two trials, were taken from the data recorded from the sensors on objects (FSR and tilt switch). This gave a resolution of 1 ms. Timings for trial 3 were taken from video data. To ensure timings were consistent for all participants, video analysis was performed by two people independently, and were tested to be within 20% tolerance, otherwise the video analysis was repeated. To ensure consistency of video capture, the camera was situated in the same position throughout the whole experiment and was not in the field of view of the participant in order to provide minimal disruption. In this experiment, the independent variables were lights, sounds and the dependent variables were time (pick up jug, spoon, pour, open drawer) and number of mistakes.

### 2.2. Trial One: Multimodal Cueing of Object State and Required Tasks

The use of simple visual cues, such as LEDs, have been shown to be robustly encoded in an action frame of reference [18], which means that the presence of an illuminated LED can be sufficient to guide a person’s attention (and reach) to that location. Interestingly, in a study of reaching to buttons cued by LEDs, patients with right stroke show improvement with practice (suggesting a beneficial effect of such cueing) while patients with left stroke do not show such improvement [19]. This suggests that, as a form of cueing, visual information, such as LEDs, could be useful for right stroke patients, but not so useful for left stroke patients. In terms of ADL, Bienkiewicz et al., showed that the noise made during the performance of a task (such as the sound of a saw, the sound of pouring water, or of stirring with a spoon) can support apraxic patients in recalling a motor program which is otherwise not accessible [20]. We presume that the effect of the cue is even stronger if the object itself emits the biological sound. For example, asking patients with Parkinson’s Disease to walk in time to the (prerecorded) sound of footsteps on gravel can lead to better support with gait problems than walking in time to a metronome [21]. This suggests that there is some element of the ‘natural’ sounds which, in addition to the marking of time, can improve performance. In terms of Tangible User Interface, early pioneers of this area referred to these as ‘graspable’ user interfaces [22], which implies that the physical form of an object would encourage physical interaction with it.

#### 2.2.1. Procedure

Participants began the experiment with both hands on the table. The experimenter then activated the handle of the jug (using the Blynk app as described previously). The jug’s handle then rose from the side of the jug. When the handle was fully raised, the LED strip on the top of the jug turned on dimly and a timer began. If the jug had not been picked up after three seconds, the LED strip turned on at full brightness. If the jug had not been picked up after six seconds, the LED strip flashed and the audio prompt of “Pick me up” was played. Once the jug had been picked up (determined by the FSR going to zero), the timer stored this time and reset. Once the participant had picked up the jug, a cup lit up and the audio prompt “pour me” was played. The timer ended when the jug was poured (tilt switch that triggered at an angle of 30°). This procedure was repeated using either the dominant or the non-dominant hand, using LED to indicate correct or distraction cups, and using two different sounds: a “pour me” voice command and the sound of the water pouring. This was done to test the effectiveness of different types of audio prompts. In terms of the ‘correct or distraction’ using LEDs, when the participant was about to perform an action with an object, another object cued the participant to use it instead. For example, when a participant was about to pour into a cup, the LED on the cup turned red and another cup turned green. This tested whether the cues can stop a current action and change an intention. At the end of the eight tasks in this trial, participants were interviewed to ask about their preference for and interpretation of the different cues that were employed, and were asked to explain what decisions they were making during the tasks.

#### 2.2.2. Results

**Figure 8:**
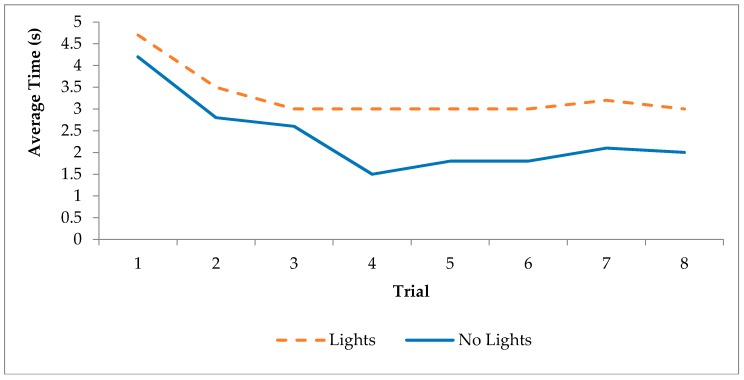
Time to complete tasks in trial one. Times for trials with and without lights are compared to a baseline condition (in which participants picked up the jug as soon as they were instructed by the experimenter).

Initially, participants would reach out to grab the jug handle, but appeared uncertain and hesitate, the “pick me up” voice then confirmed their intention. The time taken to pick up the jug was significantly quicker in the no lights case than in the lights case at trial 8 [t(19) = 1.195, *p* < 0.05] (Figure 8). The number of attempts before the “pick me up” voice was not required was not different between the lights and the no lights conditions: mean number of attempts for the lights group was 4.18 (±3.52) and in the no lights group 3.5 (±1.9). Thus, it took around 4 attempts for participants to be confident that they could pick up the jug when the handle had risen, rather than wait for the audio prompt.

All participants poured into the cup that was lit up and responded correctly to the vocal prompt. After the cup was lit, some participants would wait for the sound before beginning to pour.

None of the participants poured into an unlit cup and all the participants responded to the distraction correctly, i.e., participants stopped pouring when the light on the cup turned red and poured into another cup that turned green. There was no difference in the time taken to pour between the lights and no lights.

#### 2.2.3. Conclusions

During the post-trial debrief interviews, participants were asked what cued them to pick up the jug. In the lights case, 64% said the handle rising, 18% said the lights on the jug and 18% said the “pick me up” sound. This is in contrast with the no lights case where 90% said the handle rising and 10% said the sound cued to pick up the jug. Participants preferred the “pour me” (69%) sound than the water pouring sound (31%) as a cue to begin pouring. This was because the “pour me” is a clear instruction, whereas the water pouring was unclear. Participants preferred the water pouring sound to be after the “pour me” sound whilst they were pouring. Participants said that the water pouring sound made the experiment more realistic and indicated when they should stop pouring. When the sound stopped, all participants stopped pouring.

When the cup lit up, some participants would wait for the sound before pouring, thus the light is not strong enough as a cue to perform an action, but highlights which object the action should be performed with. Similarly, the lights did not have any effect on the number of attempts needed before the “pick me up” voice was no longer required. Without the lights, participants still picked up the jug as the animation and sound were strong cues. When participants were asked, what cued them to pick up the jug, there was less variation in the responses in the no lights case as there were fewer cues.

Change in the intensity of lights does not prompt an action. The presence of the light cued an action (as seen on the spoon and cups), but changing the brightness did not. Participants stated that when the green light appeared, it meant that an action had to be performed with the object, and they were looking for the simplest possible action. Participants also recognised that the red light meant stop pouring or do not use.

Baseline performance was also recorded. In these trials, participants were asked to complete the pick up and pour actions without waiting for cues. We found that, with the cues, Participants never reached their fastest performance when pouring. In the fastest performance, participants had a clear plan of action with clear instructions. But when cued, participants were waiting for the correct cup to light up before pouring, so there was a clear instruction and introduced a level of uncertainty. This confirms the theory that the action currently being performed is in anticipation of the next action; if the next action is unknown, then participants must wait. Another explanation is that participants were being provided with redundant information.

### 2.3. Trial Two: Hand and Handle Alignment in Picking up the Jug

We grasp tools according to the intended use [23]. Thus, while we may grasp a hammer in different ways when we want to transport it, we will grasp it at the handle with the thumb towards the heavy part when we want to use it immediately to drive a nail into a wall. Although in apraxic patients a functional grasp does not guarantee the correct use of the tool [24], such a grasp serves as a strong attractor that increases the likelihood of executing the correct gesture [25]. Even for neurotypical participants, people are faster at performing a manual response to an object when they use the hand that is aligned with the handle of a manipulable object compared to its functional end [26]. This suggests that having some means of indicating which part of an object to grasp could be useful. The action could be cued by simple modifications to the handle, e.g., by using LEDs to draw the user’s attention to the handle, or by having the handle move to indicate that it could be grasped.

Previous research has shown that people make faster responses, in reaction time tasks, when the orientation of an objects’ handle matches the hand which is to be used for the response [27,28,29,30,31,32]. Trial 2 investigated the question of whether object animation can cue an action, and whether the appearance of the handle on the jug corresponded with the hand used. Relating these tasks to the literature on task sequencing and neurological damage, when patients are asked to demonstrate perform on everyday activities (such as using a coffee machine) they can have difficulty in following multi-step procedures [33]. For patients with right brain damage, the problems related to maintaining position in a sequence of steps (i.e., they could lose track of what they had done and what they might need to do next). For patients with left brain damage, the problems were related to aphasia and retrieval of functional knowledge. This showed that performance of task sequences has different levels of impairment to those observed in the use of single objects (which often relate to difficulties in inferring use from appearance).

#### 2.3.1. Procedure

The experimental protocol was the same as for trial one, except that we only used the jug in this trial. The trials used the same participants as trial 1. Six lifts were performed testing both the dominant and the non-dominant hands. As with trial one, participants were interviewed during debrief following the tasks.

#### 2.3.2. Results

Comparison of first and sixth trial shows a significant reduction in response time: t(8) 2.962, *p* = 0.18 (with mean times reducing from 7.6 (±3.3)s on trial one to 3.6 (±2.9) s on trial six). All but 1 of the participants consistently picked up the jug with the hand to which the handle was pointing. This suggests that, for the majority of participants, handle orientation was a reliable cue as to which hand to use. Most participants found it more comfortable to use the aligned hand, and felt that the jug “asked” them to use the aligned hand, even when they might have preferred to use their dominant hand. This suggests that there is a powerful cue of the alignment of the handle. People had to be prompted at the end of the experiment to mention which hand they were picking up the jug. Some participants were using their aligned hand without being aware, as this was something they had thought about. Some said they used their dominant hand, but then they realised the alignment of the handle. One participant mentioned that they used their non-dominant hand one or twice, whereas they had used their non-dominant hand half of the time.

#### 2.3.3. Conclusions

The rising of the handle is a good affordance for determining which hand to use. Participants mentioned after the experiments that they did not notice using the other hand when picking up the jug and they felt it was natural. There was also very little difference between the timings of their non-dominant hand versus their dominant hand which explains the same reasoning.

### 2.4. Cueing Action Sequences

**Figure 9:**
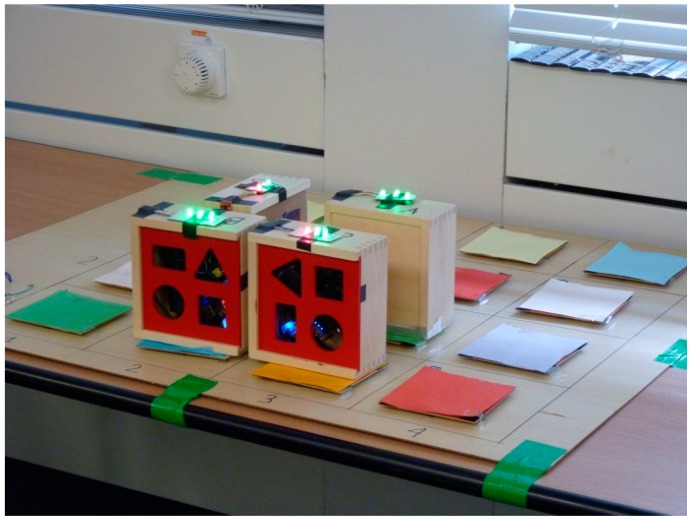
Visual feedback on networked objects [38].

In previous work, we explored the relationship between LEDs and the state of objects in a simple problem-solving exercise [33]. The objective is to ensure that four boxes had satisfied their goals (Figure 9). The goal of each box was defined by a set of rules known to the box, and defined by the position of the box and its proximity to other boxes. Each box has three LEDs representing its state: one to indicate if the goal has been satisfied, one to indicate ‘communication status’ (in terms of connection with the table), and one to indicate ‘proximity’. We were interested in whether people would try to learn the ‘rules’ that the boxes were using or whether they would find it easier to learn the pattern, or arrangement of the boxes, and whether the rules or patterns could generalize to new configurations. The argument for this comparison was that the patterns could be considered in terms of ‘affordance’ [34,35,36,37]. Not only were the patterns easier to understand (which suggests that the visual cues provides useful semantic information) but also participants found it easier to generalize patterns than the rules (contrary to our expectations).

#### 2.4.1. Procedure

The trials used the same participants as trial 1. Cues for stirring and pouring were tested with a combination of lights and sounds (from vibration motor). A sequence of actions similar to making a cup of tea was performed purely from the objects’ cues. For a ‘team making’ task, the experimenter raised the handle of the jug and, once the participant had lifted the jug, the experimenter turned the LED on one of the cups to green. When the participant had poured the jug to the cup, the experimenter turned the LED in the drawer handle green and turned on the vibration motor in the handle of the spoon (inside the drawer). The participant opened the drawer and lifted the spoon, and the experimenter turned the LED on the cup (that had been previously used to pour into) green. The participant put the spoon into the cup (perhaps making a stirring motion) and the task was completed. This constituted the ‘logical’ sequence of tea making, and was repeated four times. Additionally, an ‘illogical’ sequence was also repeated four times: this employed the same object activations but had them appear in an order that did not feel correct, e.g., taking the spoon from the drawer and stirring before pouring from the jug.

Each sequence (logical or illogical) was followed by a memory test which entailed placing cards in the same sequence that was performed. The number of mistakes was recorded and participants were not told that they will perform a memory test at the beginning the sequence. Seven cards were used: jug with handle raised, jug tilted (pouring), cup lit green, drawer opened, drawer handle lit green, spoon lit, spoon stirring cup.

Video footage from experiment three (with participant’s permission) was analysed using ELAN to obtain timings. The following timings are obtained:

- Time taken to open drawer: Timer begins when light on the handle is on and timer ends when drawer is moved.
- Time taken to pick up spoon: Timer begins when drawer fully opened and timer ends when spoon picked up.
- Time taken to stir: Timer begins when spoon is picked up till head of the spoon is inside the cup.

Midway through the experiment, only one cup lit up. There were no other audible or visual cues. In the experiment with lights on the jug, the majority of participants performed no action when the cup lit up. On the other hand, in the no lights experiment, the majority of participants picked up the jug and poured into the cup even when the jug’s handle was not raised, so participants picked up the jug with two hands and poured. As with the previous trials, participants were interviewed at debrief.

#### 2.4.2. Results

When the light on the drawer handle lit up, not all participants opened the drawer, but all participants opened the drawer after the spoon vibrated inside. There was no difference in response between a flashing drawer handle and a steady lit handle. 95% of participants stirred and all participants who did stir, stirred the cup that was lit up.

**Figure 10:**
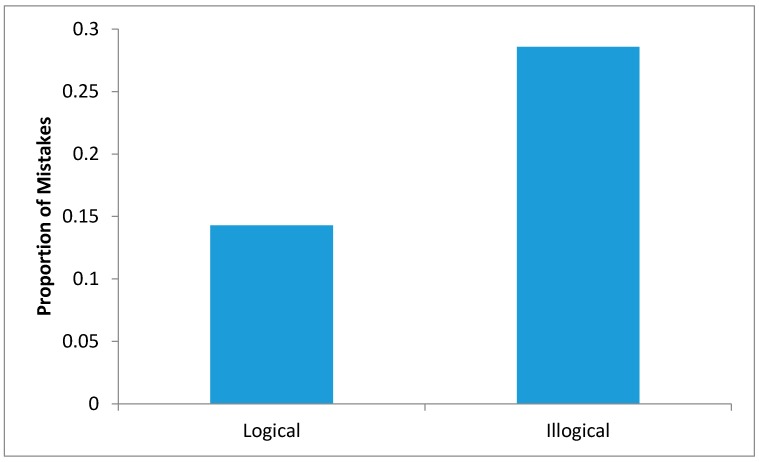
Comparing mistakes made in performing logical and illogical task sequences.

During the memory recall, participants made exactly double the number of mistakes in the illogical sequence when compared to the logical sequence (Figure 10).

There was a significant difference between the number of mistakes made in the logical and illogical sequence [t(40) = 1.118, *p* < 0.05]. Mean number of mistakes made in the logical sequence was 0.14 (±0.36) and the illogical sequence was 0.29 (±0.46).

#### 2.4.3. Conclusions

In terms of performance time, there was no significant difference between the logical and illogical sequences for all actions in the sequence (time taken to open the drawer, pick up spoon and stir). In the illogical sequence, participants found it strange to stir a cup before water was poured, but did so regardless. Flashing lights or a single steady light produced the same effect. If the participant did not respond to a light, a flashing light did not cue an action. An extra level of cueing might be needed, such as sound or animation to prompt an action. For example, a light on the drawer handle did not always cue the participant to open it, whether or not it was flashing or a steady light. However, all participants opened the drawer when the spoon was vibrating and flashing. The animation and sound of the vibration were strong enough for all participants to open the drawer. Bonanni et al. [17] claim is that if lights are placed on objects, people will always respond to the lights. However, our empirical data suggests that people will respond to the light in the context of the action being performed. People may see the light, but do not interpret it as a cue, a light does not guarantee that someone will perform an action. Sound and animation are required too to guarantee an action.

## 3. Discussion

In this paper we are considered ways in which familiar objects can be adapted to support affording situations. The overall concept is that the action that a person performs will be influenced by the context in which they perform that action. Context will, in turn, be influenced by the person’s goals, the previous tasks that they have performed, the objects available to them and the state of these objects. By recognizing the actions that the person has performed we seek to infer their goal (or intention) and from this information we modify the appearance of the objects to cue credible actions in a sequence. Future work will take the notion of cueing further so that, in addition to examining tasks in sequence we will also consider wider contextual definitions of these tasks as the basis for cueing. For example, knowing the time and that the person is walking into the kitchen, we can cue them to open a cupboard (by illuminating the cupboard handle) so that they can take out a mug (which will, once the cupboard door opens, also light up). Once the cup has been retrieved, then we can cue a sequence of steps in the preparation of a drink.

The overall goal of this work is to create, as far as practicable, subtle cues through which actions can be suggested (when this is necessary for a given user). In the case of the recovering stroke patient, the use of these cues might reduce in line with their recovery such that after a few months of using this system, there might be few or no cues presented because the patient has recovered full ability for these tasks. In the case of patients with Alzheimer’s or other forms of dementia, the cueing might need to be continued. We note, from [33], that damage to right or left brain impairs the ability to follow action sequences, and such Action Disorganisation Syndrome could be potentially aided by the concepts presented in this paper. We stress that such a claim still requires appropriate clinical testing. However, some previous work does support the idea of using multimodal cues to encourage patients to select an appropriate action in a sequence [20].

We have extended the capability of the objects designed for our prior work by incorporating actuators and displays which can provide the cues outlined in the previous sections. The overall aim is to create affording situations in which the behavior of the person can be cued by the behavior of the object which, in turn, responds to the (inferred) intentions of the user. In this case, coaching will involve building a (computer) model of the intentions of the user (based on the location of the objects, the time of day, previous activity of the person etc.) and a set of criteria for how these intentions ought to be met. The criteria could be learned by the computer or, more likely, could be defined by occupational therapists who would be working with the patient. In this case, the idea would be that a given ADL could be decomposed into specific actions, and each action could be defined in terms of quality of performance. Clearly this work is still in its infancy, and in this paper we are discussing some of the conceptual developments and initial prototypes. None of this work has been exposed to patients and thus we do not know how they might respond and react to lights, sounds or moving parts on everyday objects.

### Creating Affording Situations

In Psychology and Human-Computer Interaction, the concept of ‘affordance’ relates to the ways in which a person’s action is performed in response to their perception of the environment [34,35,36,37]. Gibson [36] introduced the term affordance into psychology, suggesting that we perceive the world in term of opportunities for action. What an object affords is determined by the physical properties of the objects (e.g., shape, orientation, size), and by the action capabilities of the agent and by the intention that the use of this object will support. Affordance effects for graspable objects can be manipulated by changing the size of the object, orientation, location and congruence. The spatial location of the handle influences stimulus identification in neglect patients, extinction patients and healthy participants. The end state comfort effect can be seen with participants having an uncomfortable grip at the beginning, then ending an action with a comfortable grip [39]. Participants who choose a grasp which was consistent with the end state comfort had a quicker reaction and movement time. Thus, an affordance is the relationship between an individual’s ability to act and the opportunities provided to that person in the given situation in pursuit of a given intention. That is, a cup of particular dimensions can be grasped by a person of particular abilities in the context of performing a task with a particular intended goal: a person with hemiparesis might struggle to use a pinch grasp on a small teacup handle; a person with tremor might find it difficult to raise a full cup to their mouth (without spillage). A person laying the table will pick up the cup differently than a person who intends to drink from it.

From this perspective, one can consider coaching in terms of the form of encouragement of, and support for, actions which are appropriate to a given context (defined by objects, person’s abilities and intentions). We aim to create ‘affording situations’ in which the appropriate action is subtly cued by the objects that the person needs to use. In this way, cueing is embedded naturally into the familiar world of the person’s home, and coaching is a matter of responding to these cues.

The manner in which a person interacts with an object can be used to infer the physical and cognitive difficulties they might be experiencing in performing the task. As the person interacts with the object, data (from sensors on the object) will be used to define the nature of the atomic tasks being performed and, from these data, the intended outcome can be inferred. Having inferred a possible outcome, objects can modify their state to invite or cue subsequent actions, or provide feedback on the task as it is performed.

The concept of affording situations is intended to highlight the importance of context in understanding affordance; it is not simply a matter of saying that a ‘jug affords lifting or pouring’. While these are actions that are, of course, possible with the jug, in order to know that this particular jug can be lifted or poured by this particular person, one also needs to know the capabilities of the person. Furthermore, in order to know whether either lifting or pouring is an appropriate action to make, one also needs to know the intention of the person (or the intention that could be plausible in that situation). Our aim is to develop technology that can recognize and interpret these contextual factors and use these to discern the plausibility of actions in a given sequence. We can then adapt the technology to provide cues that are intended to encourage the user to perform these actions. In this manner, coaching becomes a matter of creating the situations in which users of objects can learn to associate an object with an action, and an action with an intention, and an intention with a desirable outcome.

## 4. Conclusions

The objects discussed in this paper are all first-order prototypes and have been designed to support experimental evaluation with neurotypical participants, rather than patients. Patients with neurological deficit, as the result of degeneration, damage or injury, can often struggle with Activities of Daily Living (ADL). These problems can range from confusion over the sequence in which tasks should be performed, to forgetting which action can be performed using a given object, to failing to recognize a given object. These errors could involve putting breakfast cereal into a coffee mug (when making coffee) or attempting to pour from a milk container before opening it [40]. Around 1/3 of stroke survivors have difficulty with ADL and the errors that they make in planning and execution of errors can be predicted [41]. If it is possible to predict errors (rather than these being random occurrences) then it is possible to predict *when* an error could be likely to arise and to provide some cueing to either prevent the erroneous action or to encourage a preferred, i.e., non-erroneous, action.

Returning to the basic specification for this paper, for a system to provide coaching for patients with neurological deficit to allow them to perform ADL, we would need to recognize which actions people are performing (which typically means having some means of sensing that an action is being performed), to determine the quality of the performance (which requires not only activity recognition but also some evaluation of how this performance meets some criterion), to evaluate performance in terms of an anticipated outcome or intention, and to provide some means of guiding, cueing or otherwise providing advice and feedback in response to the action and in anticipation of subsequent action.

## Figures and Tables

**Table 1:** Support for coaching.

Features of Coaching	Requirements for Support
Help improve, develop, learn skills	Define goal performance
Monitor and evaluate activity	Recognise actions and predict errors
Define targets for improvement	Define measures of effectiveness
Dialogue to discuss targets and plan programme of training	Determine route from current performance to goal
Tailoring programme to individual	Modify route to cater for individual capability
Evaluate progress	Recognise action against performance goal
